# Rechargeable proton exchange membrane fuel cell containing an intrinsic hydrogen storage polymer

**DOI:** 10.1038/s42004-020-00384-z

**Published:** 2020-10-09

**Authors:** Junpei Miyake, Yasunari Ogawa, Toshiki Tanaka, Jinju Ahn, Kouki Oka, Kenichi Oyaizu, Kenji Miyatake

**Affiliations:** 1 Clean Energy Research Center, University of Yamanashi, 4-4-37 Takeda, Kofu, Yamanashi 400-8510 Japan; 2 Department of Applied Chemistry, and Research Institute for Science and Engineering, Waseda University, 3-4-1 Okubo, Shinjuku, Tokyo 169-8555 Japan; 3 Fuel Cell Nanomaterials Center, University of Yamanashi, 4-4-37 Takeda, Kofu, Yamanashi 400-8510 Japan

**Keywords:** Polymers, Fuel cells, Hydrogen storage materials

## Abstract

Proton exchange membrane fuel cells (PEMFCs) are promising clean energy conversion devices in residential, transportation, and portable applications. Currently, a high-pressure tank is the state-of-the-art mode of hydrogen storage; however, the energy cost, safety, and portability (or volumetric hydrogen storage capacity) presents a major barrier to the widespread dissemination of PEMFCs. Here we show an ‘all-polymer type’ rechargeable PEMFC (RCFC) that contains a hydrogen-storable polymer (HSP), which is a solid-state organic hydride, as the hydrogen storage media. Use of a gas impermeable SPP-QP (a polyphenylene-based PEM) enhances the operable time, reaching up to ca. 10.2 s mg_HSP_^−1^, which is more than a factor of two longer than that (3.90 s mg_HSP_^−1^) for a Nafion NRE-212 membrane cell. The RCFCs are cycleable, at least up to 50 cycles. The features of this RCFC system, including safety, ease of handling, and light weight, suggest applications in mobile, light-weight hydrogen-based energy devices.

## Abstract (web summary)

Hydrogen for fuel cells is commonly stored in pressurized tanks, whereby safety and portability can be problematic. Here, a rechargeable proton exchange membrane fuel cell with an internal hydrogen storage polymer that is cyclable up to 50 times is presented.

## Introduction

Fuel cells are promising alternative energy-converting devices that can replace fossil-fuel-based power generators^1–11^. In particular, when using hydrogen produced from renewable energy sources, the fuel cell becomes one of the cleanest possible types of power generator, since the only by-product is water. Among several types of fuel cells, proton exchange membrane fuel cells (PEMFCs) have been most successful and have already been commercialized in residential and automobile (fuel cell vehicles, FCVs) applications, owing to their high power density and efficiency at low operating temperatures (typically ca. 60–80 °C).

The fuel supply system is a crucial component in practical hydrogen PEMFC systems. Currently, a high-pressure (70 MPa) tank (three layers, glass fiber-reinforced plastic layer/carbon fiber-reinforced plastic layer/plastic liner), with a gravimetric hydrogen-storage capacity of 5.7 wt% (hydrogen weight/total weight of the tank system), is used as the state-of-the-art mode of hydrogen storage in commercial FCVs^12^; however, this system suffers from issues of volumetric hydrogen-storage capacity (or portability), safety, and energy cost. To address these issues, hydrogen-storable materials have been extensively explored^13^. For example, metal hydrides are probably the most common materials (typically, LaNi_5_H_6_)^14,15^; however, those metal hydrides generally have low gravimetric hydrogen-storage capacity. Organic hydrides, which can fix and release hydrogen chemically via covalent bonding, have also attracted significant interest (e.g., the methylcyclohexane/toluene system)^16,17^. Some of these have been claimed to simultaneously exhibit high volumetric and gravimetric hydrogen-storage capacities and have been tested for large scale hydrogen storage and transportation using the existing infrastructure for petroleum. Kariya et al. proposed a rechargeable PEMFC system using cyclohexane as the hydrogen-storage material^18^. They operated a small-scale (4 cm^2^) single cell and achieved an open circuit voltage (OCV) of 920 mV and a maximum power density of 14–15 mW cm^−2^ at 100 °C; however, this system required extra components such as a fuel reservoir, feed pump, and vaporizer, which counteracted the advantage of the high storage capacity of the organic hydrides. In addition, toxicity, flammability, and volatility were also concerns. For portable applications, longer operation time is another critical requirement. As a typical example, direct methanol fuel cells using liquid fuel were investigated for that purpose; however, they suffer from intrinsic issues such as poor safety and generation of CO, CO_2_, and other noxious gases.

**Fig. 1:**
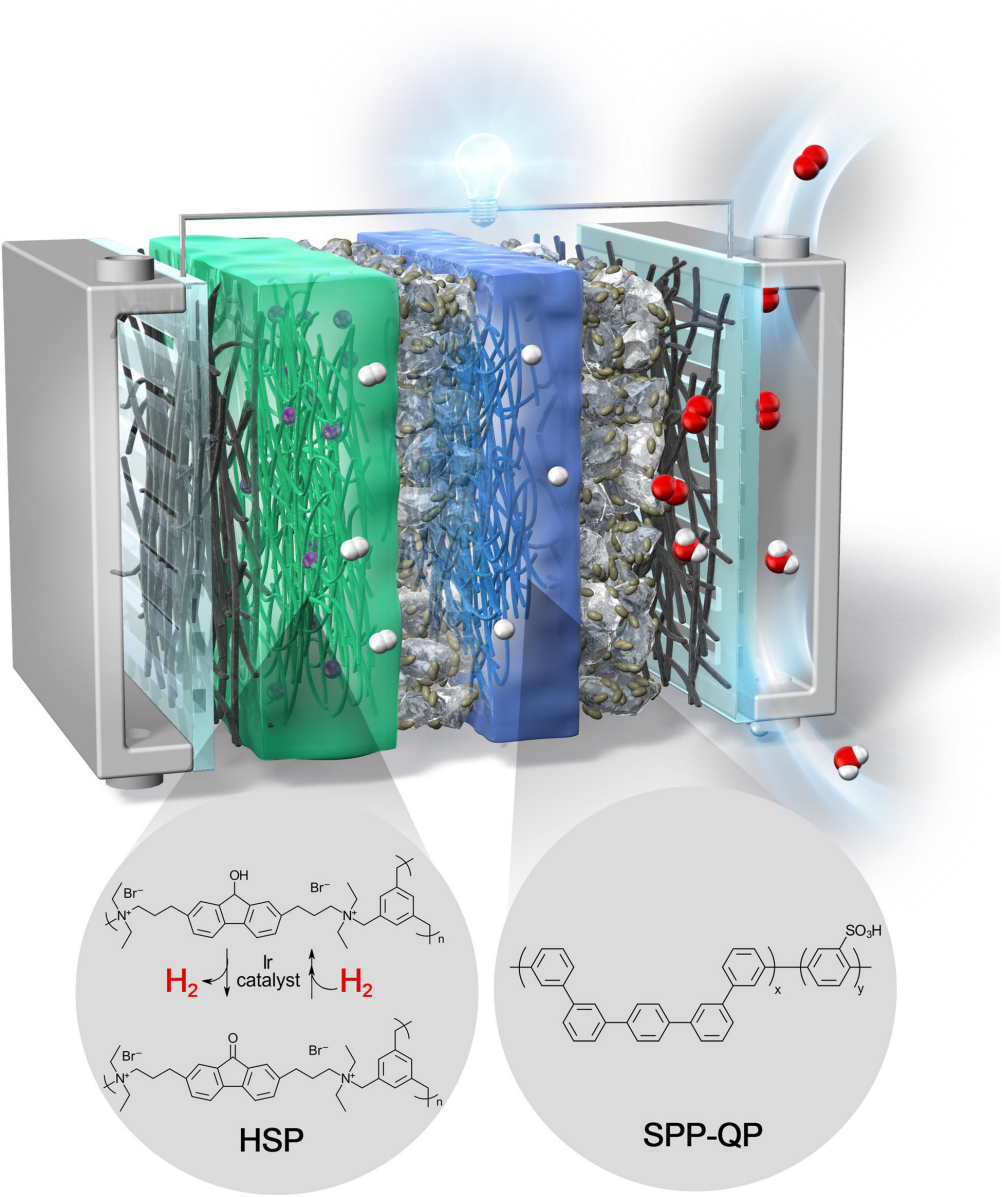
Conceptual diagram of the RCFC. An HSP sheet, which can release/fix hydrogen repeatedly, was attached onto the catalyst layer of the anode side. SPP-QP (or Nafion) membrane was used as PEM.

Recently, Kato et al. reported that a ketone/alcohol-based hydrogen-storable polymer (HSP), a solid-state organic hydride, can reversibly fix and release hydrogen with high cycleability under mild chemical reaction conditions^19^ (Fig. 1). The amorphous, nonconjugated HSP can be molded into a bendable, safe, and lightweight sheet form, and the HSP sheet can stably fix hydrogen at room temperature and ambient pressure and release hydrogen at 80 °C. This feature is highly suitable for a rechargeable PEMFC, in which an HSP sheet can be easily mounted into the anode side of the cell. Herein, we propose an “all-polymer type” rechargeable PEMFC system, by applying the HSP sheet as a hydrogen-storage medium inside the cell, which neither requires pressurized hydrogen tank nor cumbersome metal hydrides.

## Results and discussion

### Design of the rechargeable fuel cell (RCFC)

**Fig. 2:**
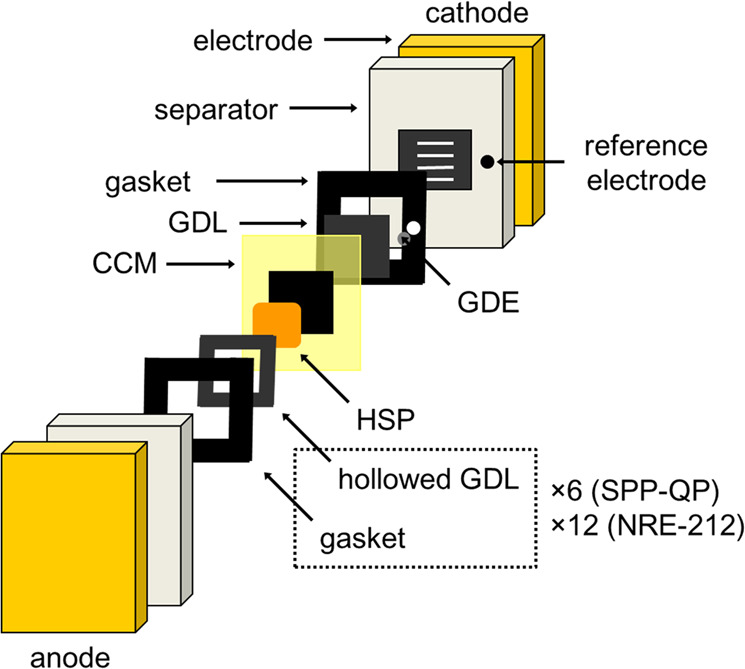
Configuration of the membrane electrode assembly (MEA) for the RCFC. CCM and GDL refer to catalyst-coated membrane and gas diffusion layer, respectively. Number of GDLs and gaskets in the anode side differed depending on the thickness of HSP used in each experiment.

Figure 1 represents the conceptual diagram of the RCFC. The HSP sheet as a hydrogen-releasing/fixing media was attached onto the catalyst layer (CL) of the anode side. An Ir catalyst (aqua(6,6′-dihydroxy-2,2′-bipyridine)(pentamethylcyclopentadienyl)iridium(III) bis(triflate)^20^) was loaded inside the HSP sheet to facilitate the hydrogen-releasing/fixing reactions. Figure 1 also includes the scheme with the detailed chemical structures of the HSP^19^. In the structure, the fluorenol/fluorenone groups have the hydrogen-storage functionality. Due to the network (cross-linked) structure with quaternary ammonium groups, the HSP, either in the fluorenol or fluorenone forms, was not soluble in water but became swollen with water to form a hydrogel. The HSP sheet released 20%, 33%, 51%, or 96% of the total fixed hydrogen gas in 20, 30, 60, or 360 min, respectively, at 80 °C in the presence of the Ir catalyst under wet conditions (Supplementary Fig. 1). The Ir catalyst could also absorb up to 58 mol% hydrogen at 1 atm of H_2_, which was substantially lower (ca. 4.7 wt%) than that stored in HSP. Figure 1 further shows the chemical structure of the PEM (SPP-QP) used in this study^10^. The SPP-QP, which we have recently developed, is a fluorine-free, fully-aromatic-type PEM, whose gas barrier properties are far superior to that of a commercially available, perfluorinated-type PEM such as Nafion. Hydrogen and oxygen gas permeabilities of SPP-QP (ion exchange capacity (IEC) of 2.4 mmol g^−1^) at 80 °C and 90% relative humidity (RH) were 1.46 × 10^−9^ and 4.72 × 10^−10^ cm^3^ (STD) cm cm^−2^ s^−1^ cmHg^−1^, respectively, compared to those (7.35 × 10^−9^ and 3.15 × 10^−9^ cm^3^ (STD) cm cm^−2^ s^−1^ cmHg^−1^) of a Nafion NRE-212 membrane. In addition, the SPP-QP membrane fulfills other required properties for fuel cell applications in terms of proton conductivity and stability (e.g., thermal/mechanical/chemical). RCFC performance is compared between SPP-QP and Nafion NRE-212 cells in details. Figure 2 shows the detailed configuration of the membrane electrode assembly (MEA) used in the present study. For the cathode side, the MEA configuration is the same as that of a normal PEMFC^10^. For the anode side, a porous gas diffusion layer (GDL) was used. To adjust the thickness with the HSP sheet (note that HSP was 1.5–3.3-mm thick), multiple GDLs and gaskets were used to ensure tight seal.

### Protocol for the RCFC evaluation

**Fig. 3:**
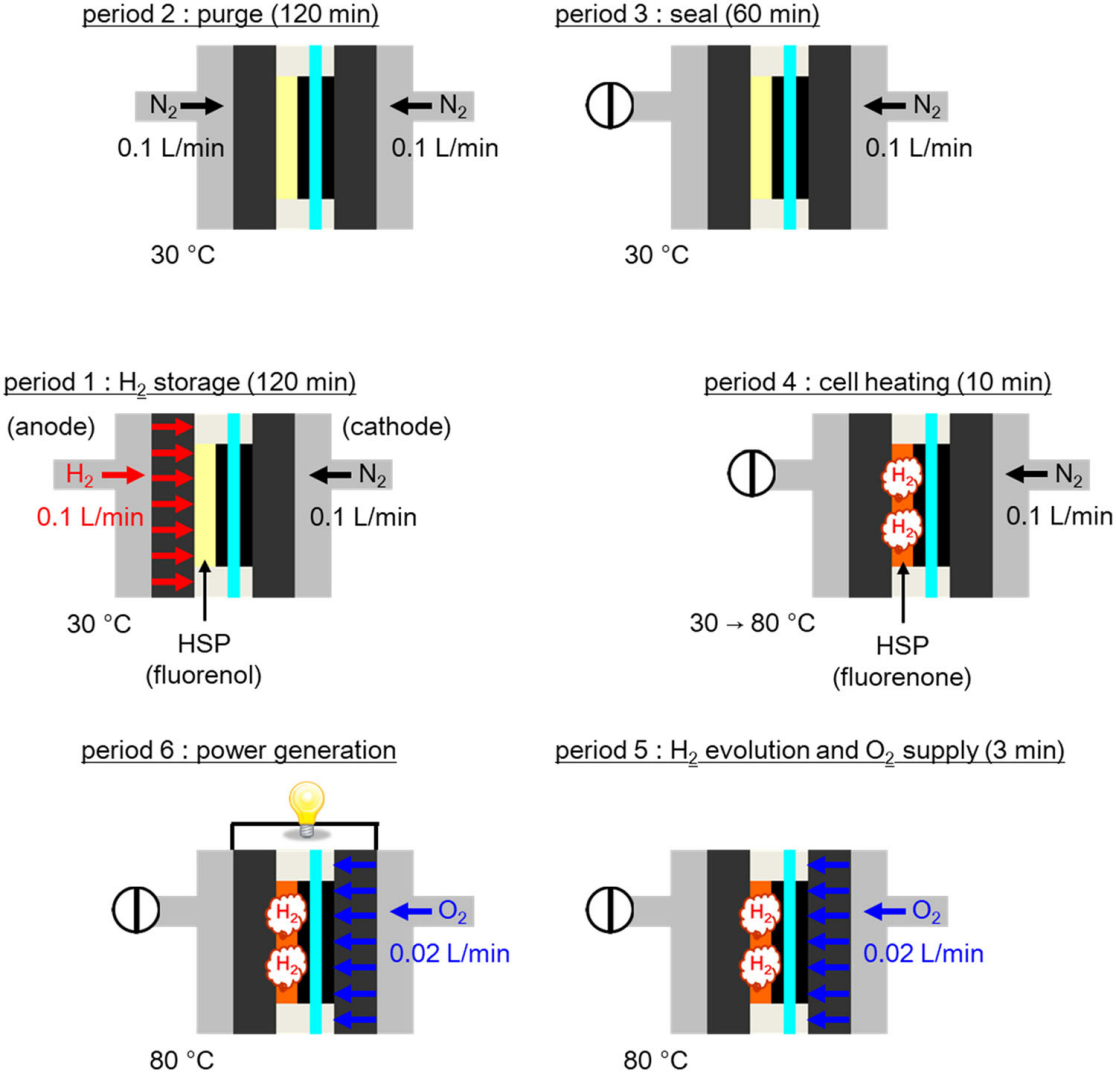
Flowchart of the RCFC evaluation protocol. The RH was set at 100% in all cases.

Figure 3 represents the flowchart of the RCFC evaluation protocol. The humidity was always set at 100% RH (relative humidity) for efficient hydrogen-releasing/fixing reactions of the HSP in the presence of water. During time period 1, hydrogen was infused into the HSP sheet by supplying hydrogen gas to the anode at 30 °C for 120 min. During period 2, nitrogen gas was purged to the anode to flush the hydrogen gas from the anode. During period 3, the anode side was sealed. During period 4, the cell was heated to 80 °C for 10 min to initiate the hydrogen release from the HSP sheet. During period 5, oxygen gas was supplied to the cathode for 3 min without power generation. During period 6, the fuel cell was operated at a constant current density. This protocol was repeated to investigate the cycle performance and durability of the RCFC.

### Fuel-cell performance

Prior to the detailed fuel-cell evaluation with our SPP-QP membrane, preliminary experiment was conducted with a commercially available Nafion NRE-211 membrane (25-μm thick). The NRE-211 cell was operable only for ca. 14 s at a constant current density of 10 mA cm^−2^ with 44.7 mg of HSP (Supplementary Fig. 2). To increase the operation time, the membrane was replaced with a Nafion NRE-212 (50-μm thick), and larger amount of HSP (122.5 mg) was used. The cell was operable for ca. 17 s but still rather short in spite of the thicker membrane and larger amount of hydrogen source. We speculated that use of SPP-QP as gas impermeable polyphenylene-based PEM must enhance the operable time. Comparison of fuel-cell performance is made for Nafion NRE-212 and SPP-QP cells hereafter.

During the O_2_ supply for 3 min (before initiation of power generation) during period 5, the cell voltage, anode and cathode potentials, and ohmic resistance were monitored as a function of the O_2_ supply time (Supplementary Fig. 3). In the Nafion NRE-212 cell, the cell voltage was initially 0.742 ± 0.040 V, and increased to 0.820 ± 0.034 V after 180 s. The cathode potential was closely linked to the cell voltage; i.e., increased from 0.837 ± 0.022 to 0.921 ± 0.009 V, suggesting that the increase in the cell voltage mainly resulted from the O_2_ diffusion in the cathode. Although the O_2_ filling in the cathode was not complete and the OCV was lower than that (≈1.0 V) expected for a typical H_2_/O_2_ PEMFC, we chose not to prolong the flow of O_2_ to avoid unfavorable O_2_ cross-over to the anode, with inevitable consumption of stored hydrogen. The ohmic resistance decreased slightly with time, from 0.037 ± 0.004 to 0.035 ± 0.003 mΩ cm^2^, due to the lower flow rate (20 mL min^−1^) of O_2_ (to cause higher hydration level of the membrane) than that of N_2_ (100 mL min^−1^ during period 4). The anode potential increased only slightly with time, from 0.097 ± 0.029 to 0.102 ± 0.028 V (Supplementary Fig. 3c inset, for clarity) in spite of the continuous H_2_ evolution during this time. It is likely that some loss of H_2_ might have occurred, either by permeating to the cathode or by oxidation with O_2_ permeated from the cathode. In contrast, in the SPP-QP cell, the anode potential was lower and decreased slightly with time, from 0.074 ± 0.014 to 0.072 ± 0.013 V (Supplementary Fig. 3d inset, for clarity). This is indicative of a smaller loss of H_2_ in the anode of the SPP-QP cell due to the much lower gas permeability of the SPP-QP membrane in comparison with that of the Nafion NRE-212 membrane, as mentioned above. The H_2_ filling was not up to the highest obtainable level in the present protocol, as suggested by the anode potential (0.072 ± 0.013 V), even for the SPP-QP cell, compared to that (≈ 0 V) of a typical H_2_/O_2_ PEMFC.

**Fig. 4:**
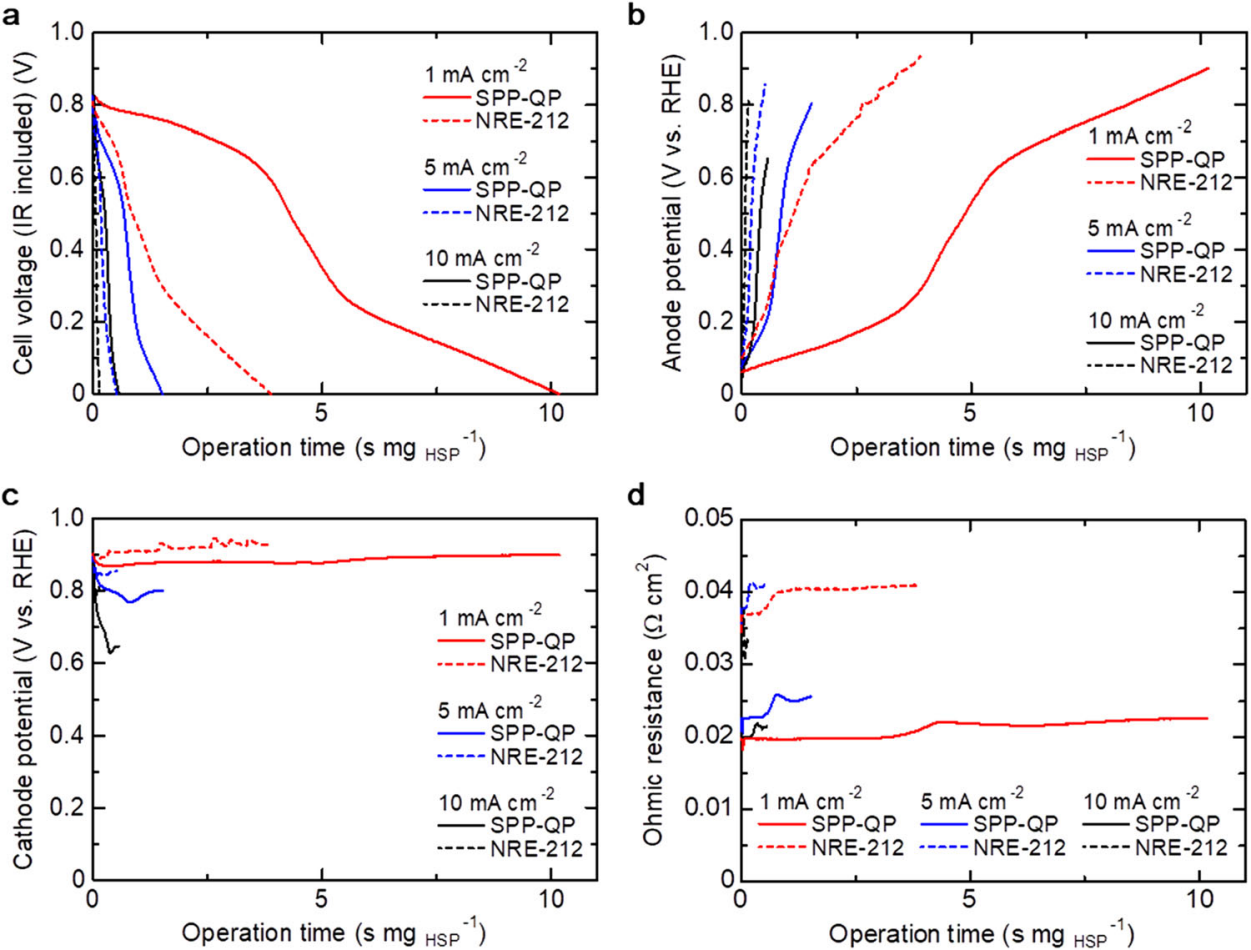
Fuel-cell performance at a constant current density of 1, 5, 10 mA cm^−2^ (period 6, Fig. 3). **a** Cell voltage, **b** anode potential, **c** cathode potential, and **d** ohmic resistance as a function of operation time, which is normalized by HSP weight. The fuel cells were operated at 80°C and 100% RH, in which the flow rate of O_2_ was 20mLmin^−1^.

After the O_2_ supply for 3 min, the fuel-cell operation (i.e., power generation) was started (period 6, Fig. 3). Figure 4 represents the cell voltage, anode and cathode potentials, and ohmic resistance at a constant current density of 1, 5, 10 mA cm^−2^ as a function of the operating time (see Supplementary Fig. 4, for iR-free cell voltage). Note that the operating time is normalized by HSP weight for the quantitative understanding of the effect of the different membranes. The OCVs of the Nafion NRE-212 and SPP-QP cells were 0.81 and 0.83 V, respectively, somewhat low for an H_2_/O_2_ PEMFC due to insufficient H_2_/O_2_ filling, as discussed above. At a constant current density of 10 mA cm^−2^, the Nafion NRE-212 cell was operable for ca. 0.15 s mg_HSP_^−1^ (or ca. 18 s for 123 mg of HSP). As shown in Fig. 4b (Supplementary Fig. 5, for clarity), the anode potential of the Nafion NRE-212 cell immediately increased, while the cathode potential was nearly constant, indicating that the H_2_ consumption exceeded the H_2_ supply from the beginning. In contrast, the anode potential of the SPP-QP cell maintained a low value for the initial ca. 0.2 s mg_HSP_^−1^, indicating that H_2_ released from the HSP sheet was sufficient for power generation at a constant current density of 10 mA cm^−2^ for this period. After this time period, the anode potential increased (but still more slowly than that of the Nafion NRE-212 cell), because the H_2_ supply was not able to match the H_2_ consumption. Consequently, the SPP-QP cell was operable for ca. 0.56 s mg_HSP_^−1^ (or ca. 28 s for 50 mg of HSP), which was ca. four times longer compared with that (ca. 0.15 s mg_HSP_^−1^ (or ca. 18 s for 123 mg of HSP)) of the Nafion NRE-212 cell. This is again because of the much lower H_2_ permeability of the SPP-QP membrane in comparison with that of the Nafion NRE-212 membrane. Since SPP-QP was thinner and more proton-conductive, the ohmic resistance of the SPP-QP cell was ca. 21 mΩ cm^2^, i.e., ca. 48% lower than that of the Nafion NRE-212 cell (Fig. 4d).

Then, effect of the current density was investigated. As shown in Fig. 4a, the operable time increased with decreasing current density (from 10–5 to 1 mA cm^−2^), and the effect was much more pronounced for the SPP-QP cell than for the Nafion NRE-212 cell (see also Supplementary Fig. 6). The maximum operable time was observed for the SPP-QP cell at a constant current density of 1 mA cm^−2^ and reached ca. 10.2 s mg_HSP_^−1^ (or ca. 509 s for 50 mg of HSP), which was more than a factor of two longer than that (3.90 s mg_HSP_^−1^ (or 478 s for 123 mg of HSP)) for the Nafion NRE-212 cell under the same conditions. The anodic overpotential increased more slowly with decreasing current density because of the slower H_2_ consumption at lower current density (Fig. 4b). The cathodic overpotential similarly increased more slowly with decreasing current density for the same reason (Fig. 4c).

**Fig. 5:**
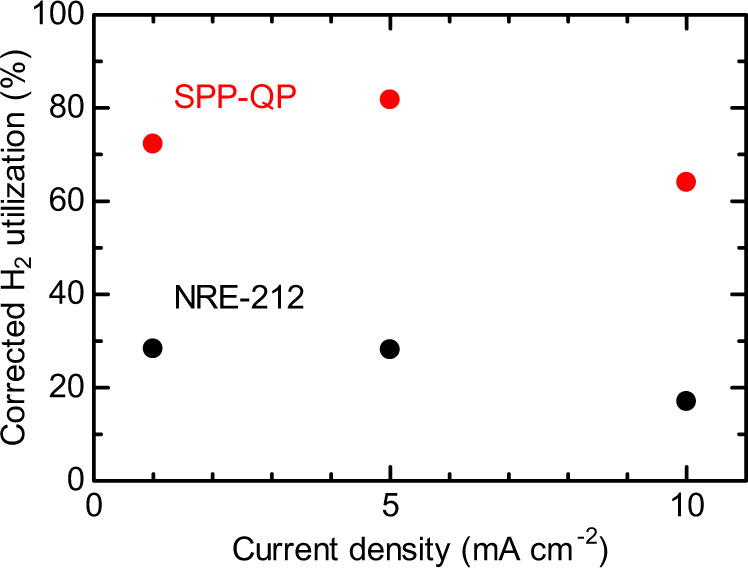
Corrected H_2_ utilization at a constant current density of 1, 5, 10 mA cm^−2^. The corrected H_2_ utilization was defined as experimentally generated electricity/theoretically obtainable electricity. The theoretically obtainable electricity was calculated based on the fixed H_2_ in the HSP sheet and the H_2_ yield (or conversion, *h*) calculated by Eq. 1.

The H_2_ utilization value, defined as experimentally generated electricity/theoretically obtainable electricity calculated from the stored H_2_ in the HSP sheet, was relatively low, 5.8% for the Nafion NRE-212 cell and 15% for the SPP-QP cell at 1 mA cm^−2^, decreasing to 2.2% for Nafion NRE-212 and 8.3% for SPP-QP at 10 mA cm^−2^ (Supplementary Fig. 7). The utilization was low for both cells mainly due to the slow kinetics of the H_2-_releasing reaction of the HSP sheet. Since the operation time was much shorter than the time required for the full release of the stored H_2_ (Supplementary Fig. 1), a corrected H_2_ utilization is defined, which is based on the amount of H_2_ actually released, calculated from the estimated H_2_ evolution time using the following first-order reaction kinetic equation:

1 $$- {\mathrm{ln}}\frac{{\left[ {{\mathrm{Fluorenol}}\;{\mathrm{polymer}}} \right]}}{{\left[ {{\mathrm{Fluorenol}}\;{\mathrm{polymer}}} \right]0}} = - \ln \left( {1 - {h}} \right) = {kt},$$

where *h* is the H_2_ yield (or conversion), *k* is the reaction rate coefficient, and *t* is the estimated H_2_ evolution time (corresponding to time periods 4–6 in Fig. 3). The corrected H_2_ utilization is plotted as a function of the current density in Fig. 5.

The corrected H_2_ utilization showed a unique dependence on the current density. For both cells, the utilizations were minima at the current density of 10 mA cm^−2^ (17.0% for Nafion NRE-212 and 64.0% for SPP-QP), and increased (to 28.1% for Nafion NRE-212 and 81.7% for SPP-QP) at 5 mA cm^−2^, then nearly saturated (28.3% for Nafion NRE-212) or decreased (72.2% for SPP-QP) at 1 mA cm^−2^. At 1 mA cm^−2^, gas permeation through the membrane might have also affected the operable time. Nevertheless, the SPP-QP cell exhibited much higher H_2_ utilization than that of the Nafion NRE-212 cell at any current density.

**Fig. 6:**
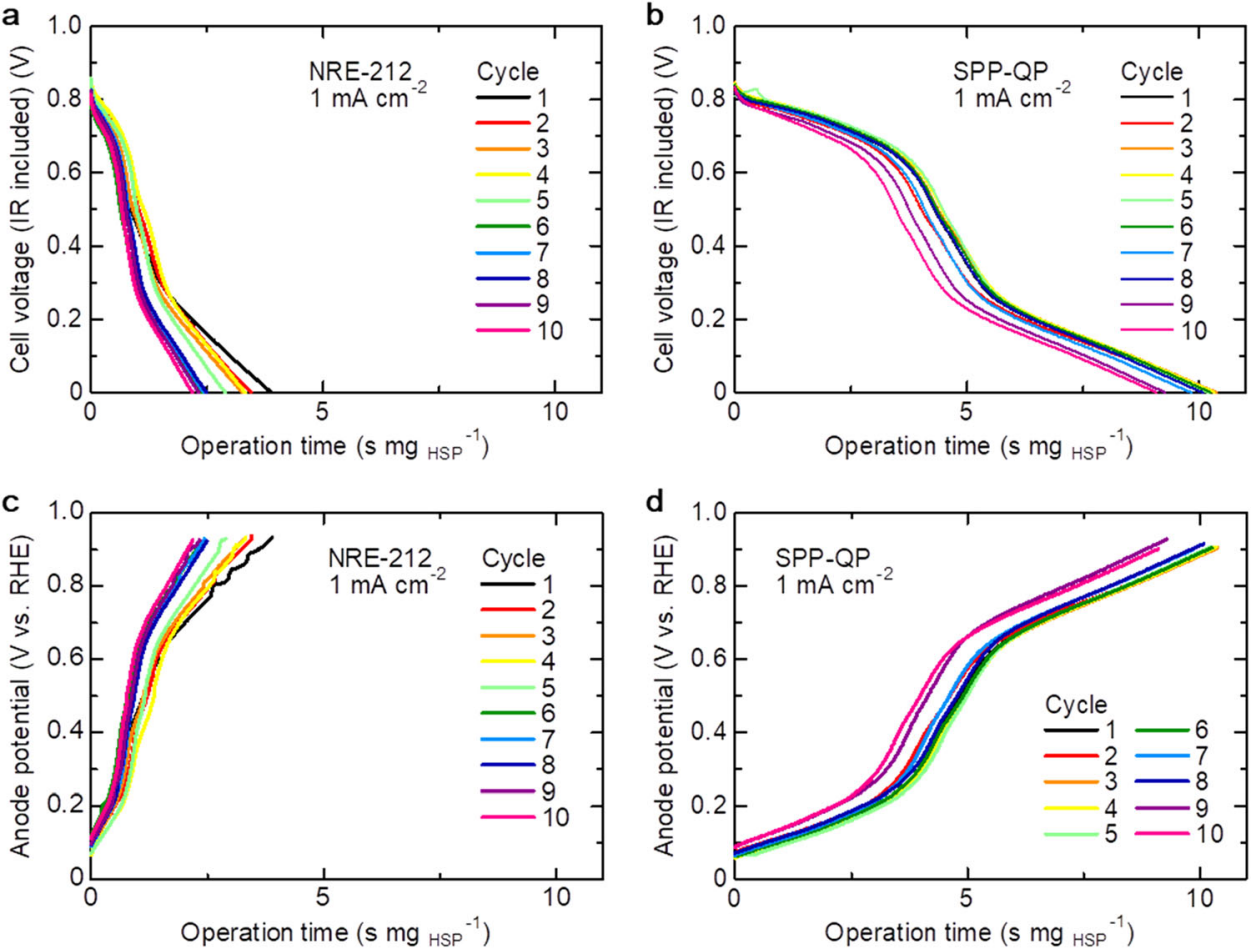
Cycle dependence of the RCFC performance at a constant current density of 1 mA cm^−2^. **a**, **b** Cell voltage and **c**, **d** anode potential as a function of operation time normalized by HSP weight. The fuel cells were operated at 80 °C and 100% RH, in which O_2_ flow rate was 20 mL min^−1^.

**Fig. 7:**
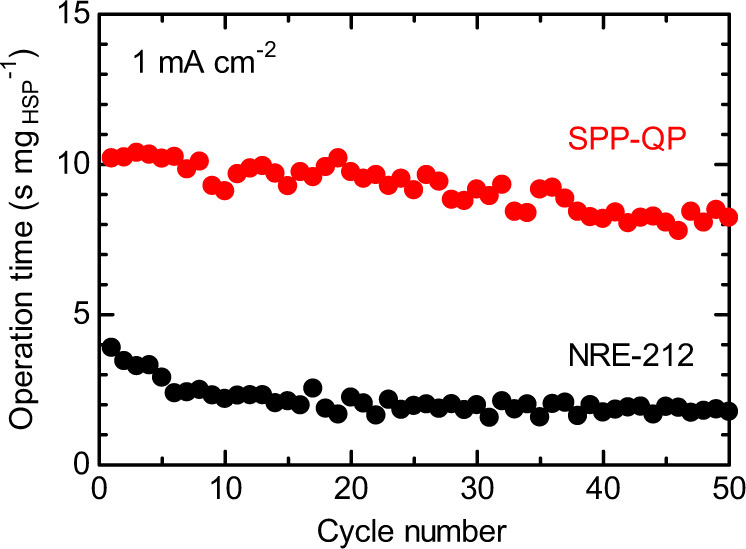
Cycle performance of the RCFC at a constant current density of 1 mA cm^−2^. The RH was set at 100% throughout the experiments.

Figure 6a, b represents the cycle performance of the RCFC at the current density of 1 mA cm^−2^. Both cells were operable with cycleability at least up to 50 cycles (Fig. 7). During the cycling, the SPP-QP cell exhibited 6–7 s mg_HSP_^−1^ longer operation time than that of the Nafion NRE-212 cell. In both cells, however, the operable time gradually decreased with cycling. With increasing number of cycles, the anodic overpotential increased (Fig. 6c, d), while the changes in the cathode potential and ohmic resistance were rather minor (Supplementary Fig. 8), indicating that the amount of H_2_ released from the HSP sheet might have gradually decreased with increasing number of cycles.

After 50 cycles, the RCFCs were disassembled, and the recovered HSP sheets were subjected to post-test analyses (Supplementary Fig. 9). In the ^1^H and ^13^C NMR spectra, practically no changes were detected in the polymer structure, while loss of the bipyridine ligand of the Ir catalyst was confirmed. In the ^1^H NMR spectra of the Ir catalyst, appearance of unknown signals was also confirmed. The results indicate that the deterioration of the RCFC with cycling resulted from the leaching and/or the decomposition of the Ir catalyst under the RCFC conditions. Searching the further robust catalyst for the reversible hydrogenation of HSP is the topic of our continuing research.

## Conclusion

We have demonstrated the principle and cycle performance of the “all-polymer type” rechargeable PEMFC (RCFC). The use of a low gas permeability PEM, SPP-QP (a polyphenylene-based PEM), was a crucial strategy to enhance the operable time of the RCFC. The maximum operable time was observed for the SPP-QP membrane cell at a constant current density of 1 mA cm^−2^ and 80 °C, and reached ca. 10.2 s mg_HSP_^−1^ (or ca. 509 s for 50 mg of HSP), which was more than a factor of two longer than that (3.90 s mg_HSP_^−1^ (or 478 s for 123 mg of HSP)) for the Nafion NRE-212 membrane cell under the same conditions. The RCFCs exhibited reasonable cycleability, at least up to 50 cycles. The mechanistic study herein indicated that the H_2_ storage capacity and kinetics (H_2-_releasing/fixing reactions) of the HSP and stability of the catalyst need improvement for further enhancement of the RCFC performance and cycle durability. The features of the RCFC, including safety, ease of handling, and lightweight, could lead to a paradigm shift in mobile PEMFC applications.

## Methods

### Materials

The Ir catalyst (aqua(6,6′-dihydroxy-2,2′-bipyridine)(pentamethylcyclopentadienyl)iridium(III) bis(triflate)^20^, Kanto Chemical) was used as received. The SPP-QP membrane^10^ (25-μm thick, IEC = 2.47 mmol g^−1^) and the HSP sheet^19^ (containing 12.6 wt% of the Ir catalyst) were prepared according to the literature.

### Catalyst paste preparation

A catalyst paste was prepared by mixing a commercial Pt/CB catalyst (1 g, TEC10E50E, Tanaka Kikinzoku Kogyo K. K.), 5 wt% Nafion dispersion (7.52 g, IEC = 0.95–1.03 mmol g^−1^, D-521, Du Pont), deionized water (4.19 g), and ethanol (8.21 g) by ball milling with zirconia balls (*φ* = 5 mm) using planetary ball mill (PULVERISETTE 6, FRITSCH) at 270 rpm for 30 min. The mass ratio of the Nafion binder to the carbon support (N/C) was adjusted to 0.70.

### MEA fabrication

The SPP-QP cell was prepared as follows. The catalyst-coated membrane (CCM) was prepared by spraying the catalyst paste on both sides of the SPP-QP membrane (25-μm thick, IEC = 2.47 mmol g^−1^) by means of the pulse-swirl-spray machine (Nordson, nozzle type: A7A for Swirl ver. 3 Dual syringe, type2). The CCM was dried at 60 °C for 12 h and hot-pressed (in-house hot press machine) at 140 °C and 10 kgf cm^−2^ for 3 min. The geometric area and the Pt-loading amount of the CL were 4.41 cm^2^ and 0.50 ± 0.05 mg cm^−2^, respectively. For the cathode side, a GDL (29BC, SGL Carbon Group Co., Ltd, 230-μm thick) and a gasket (silicon rubber–polyethylene naphthalate–silicon rubber (Maxell Kureha Co., Ltd), 200-μm thick, quadratic prism (ca. 6 × 6 cm^2^) hollowed out (ca. 2.1 × 2.1 cm^2^) in the center), and for the anode side, a HSP sheet (50 mg, square frustum [1.5-mm thick, upper base ca. 1 × 1 cm^2^, lower base ca. 2 × 2 cm^2^]), six GDLs (the same GDL as the above, but hollowed out (ca. 2 × 2 cm^2^) in the center, 1.38-mm thick in total), and six gaskets (the same gasket as above, 1.2-mm thick in total) were placed on the CCM and mounted into a cell that had serpentine flow channels on both the anode and the cathode carbon separators. The Nafion NRE-212 cell using Nafion NRE-212 membrane (50-μm thick, IEC = 0.98 mmol g^−1^, Du Pont) was prepared in a similar manner; an HSP sheet (122.5 mg, square frustum [3.3-mm thick, upper base ca. 1 × 1 cm^2^, lower base ca. 2 × 2 cm^2^]), twelve GDLs (the same GDL as the above, but hollowed out (ca. 2 × 2 cm^2^) in the center, 2.76-mm thick in total), and twelve gaskets (the same gasket as above, 2.4-mm thick in total) were used for the anode side.

## Supplementary information

Supplementary Information

Peer Review File

## Data Availability

The data that support the findings of this study are available from the corresponding author upon reasonable request.
