# Supplementary material for: Rechargeable proton exchange membrane fuel cell containing an intrinsic hydrogen storage polymer
Source: Commun Chem. 2020 Oct 9;3:138. doi: 10.1038/s42004-020-00384-z (PMC9814259; doi:10.1038/s42004-020-00384-z)
Supplement: Supplementary file 1 — Supplementary Information [file 42004_2020_384_MOESM1_ESM.docx]

*SUPPLEMENTARY INFORMATION*

**Rechargeable proton exchange membrane fuel cell containing an intrinsic hydrogen storage polymer**

Junpei Miyake^1^, Yasunari Ogawa^1^, Toshiki Tanaka^1^, Jinju Ahn^1^, Kouki Oka^2^, Kenichi Oyaizu^2^ & Kenji Miyatake^1,2,3^*

*^1^Clean Energy Research Center, University of Yamanashi, 4-4-37 Takeda, Kofu, Yamanashi 400-8510, Japan.*

*^2^Department of Applied Chemistry, and Research Institute for Science and Engineering, Waseda University, 3-4-1 Okubo, Shinjuku, Tokyo 169-8555, Japan.*

*^3^Fuel Cell Nanomaterials Center, University of Yamanashi, 4-4-37 Takeda, Kofu, Yamanashi 400-8510, Japan.*

*Corresponding author

Email: miyatake@yamanashi.ac.jp

**SUPPLEMENTARY FIGURES**

**Supplementary Figure 1 |** H_2_ yield as a function of time. **a**,**b** The HSP sheet (10 mg mL^-1^ of Ir catalyst (triflate)) was heated at 80 °C in air. The H_2_ releasing reaction was monitored by FT-IR spectra, in which the H_2_ yield (or conversion, h) was quantified by the C=O stretching vibration peak (ca. 1710 cm^-1^). The kinetics of the reaction was estimated by the following equation.

$$-ln\frac{\left[ fluorenol polymer \right]}{\left[ fluorenol polymer \right]0}=-ln \left( 1-h \right)= kt$$

**Supplementary Figure 2 |** Fuel cell performance at a constant current density of 10 mA cm^-2^. The RCFC was monitored at 80 °C and 100% RH, with flowing O_2_ at 20 mL min^-1^. The membrane thickness and loading amount of HSP were 25 μm and 44.7 mg for Nafion NRE-211 cell and 50 μm and 122.5 mg for Nafion NRE-212 cell, respectively.


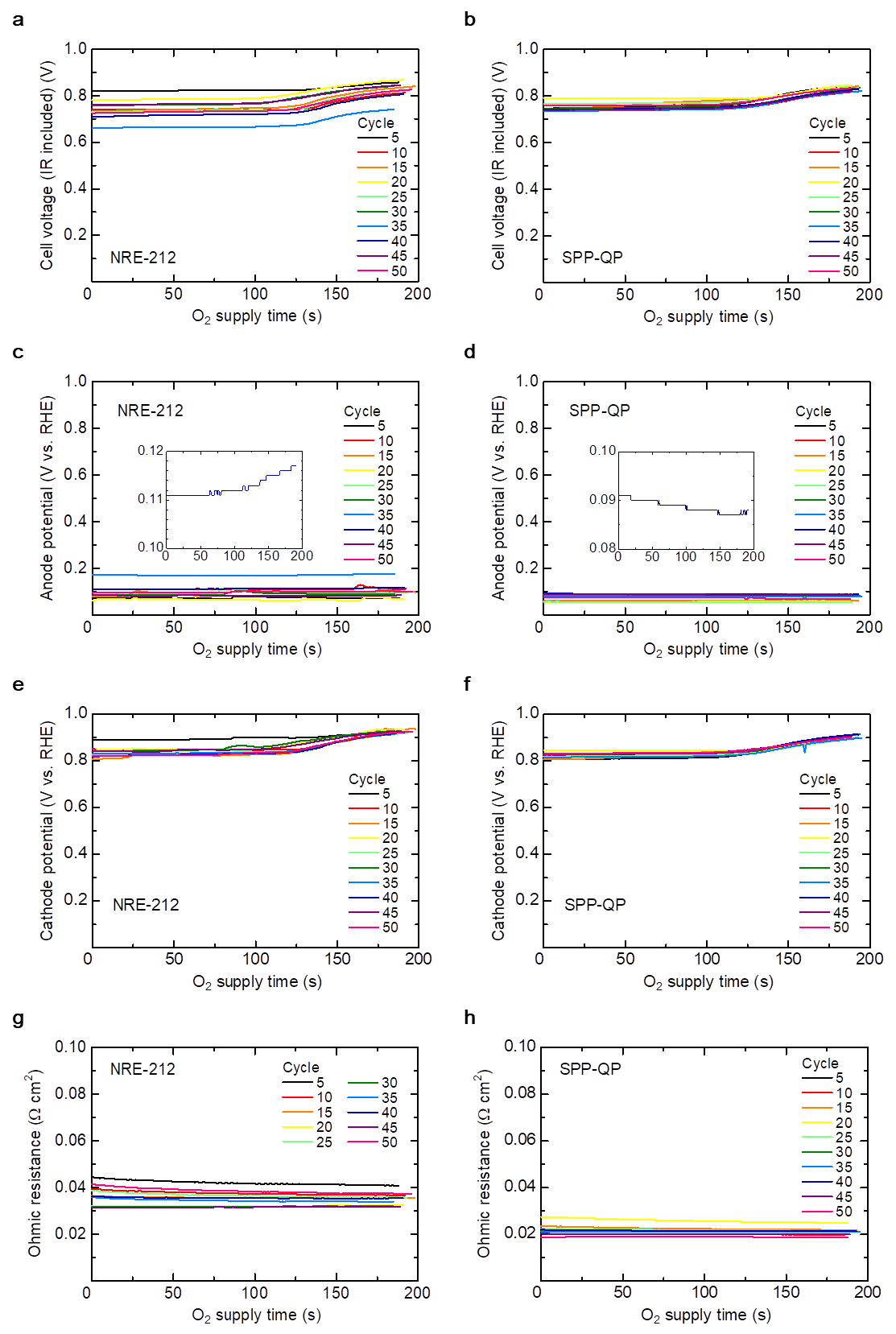


**Supplementary Figure 3 |** Before fuel cell operation (period 5, Fig. 3). **a**,**b** Cell voltage, **c**,**d** anode potential, **e**,**f** cathode potential, and **g**,**h** ohmic resistance as a function of O_2_ supply time. The RCFC was monitored at 80 °C and 100% RH, with flowing O_2_ at 20 mL min^-1^.

**Supplementary Figure 4 |** IR free representation of Fig. 4a (period 6, Fig. 3). Cell voltage as a function of operation time, which is normalized by HSP weight. The fuel cells were operated at 80 °C and 100% RH, in which the flow rate of O_2_ was 20 mL min^-1^.

**Supplementary Figure 5 |** Magnification of Fig. 4b (period 6, Fig. 3). Anode potential (80 °C and 100% RH) as a function of operation time normalized by HSP weight. The fuel cells were operated at a constant current density of 10 mA cm^-2^, in which the O_2_ flow rate was 20 mL min^-1^.

**Supplementary Figure 6 |** Operable time at a constant current density of 1, 5, 10 mA cm^-2^.

**Supplementary Figure 7 |** H_2_ utilization at a constant current density of 1, 5, 10 mA cm^-2^. The H_2_ utilization was defined as experimentally generated electricity / theoretically obtainable electricity calculated from the fixed H_2_ in the HSP sheet.

**
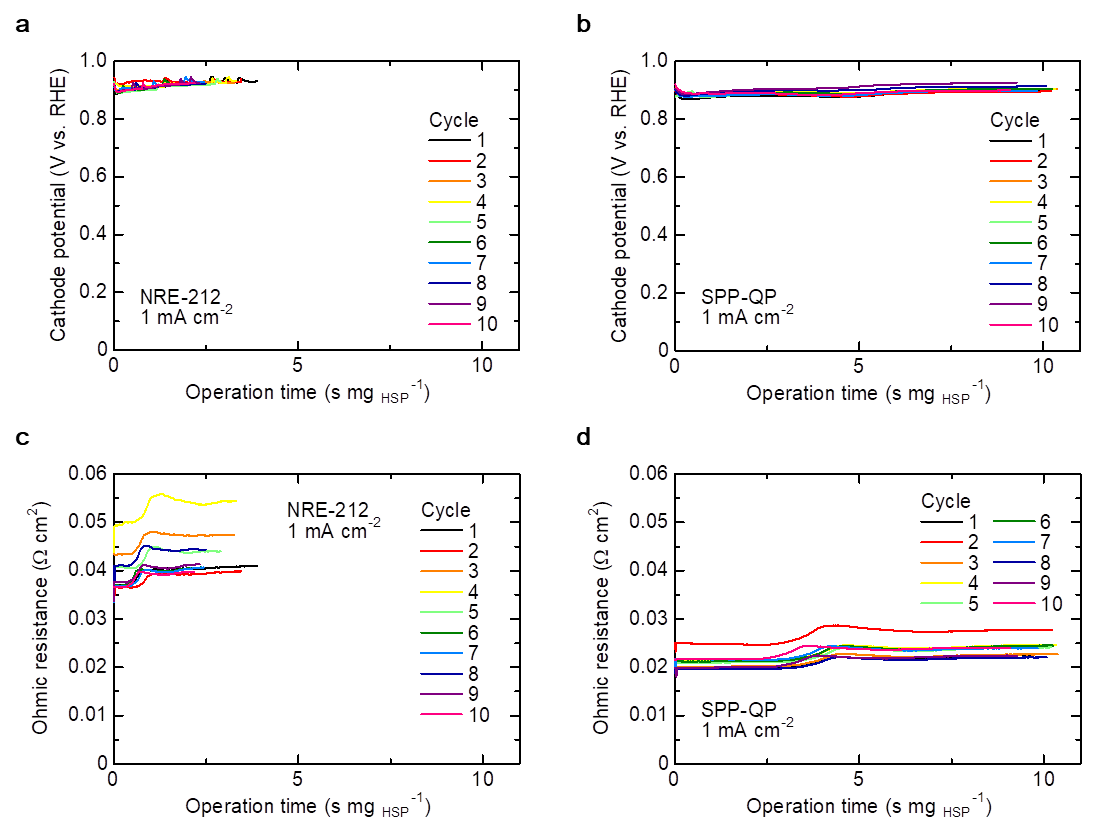
Supplementary Figure 8 |** Cycle dependence of the RCFC performance at a constant current density of 1 mA cm^-2^. **a**,**b** Cathode potential and **c**,**d** ohmic resistance as a function of operation time normalized by HSP weight. The fuel cells were operated at 80 °C and 100% RH, in which O_2_ flow rate was 20 mL min^-1^.


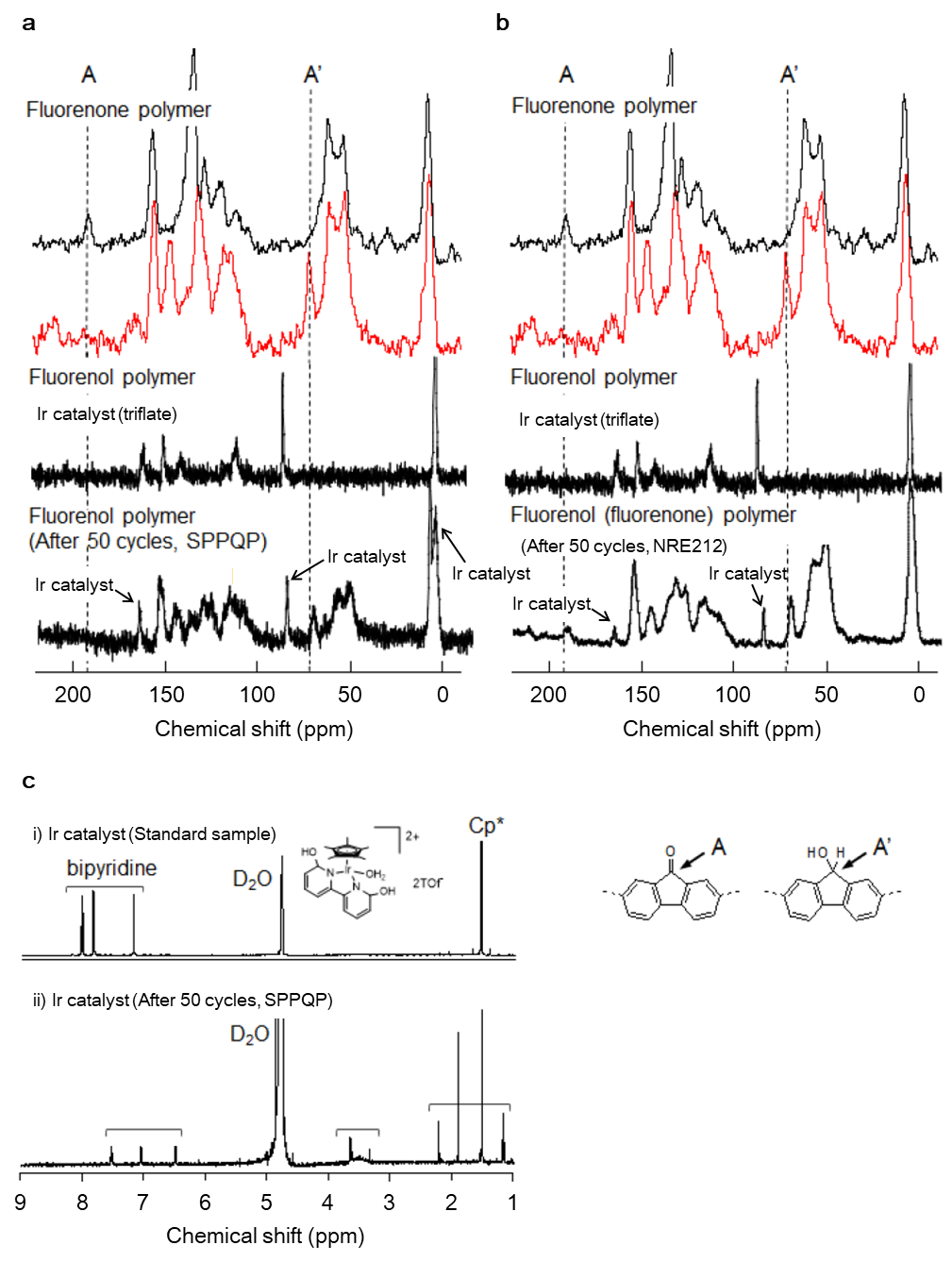


**Supplementary Figure 9 |** Post-test analyses of HSP sheet after 50 RCFC cycles. **a**,**b** Solid-state ^13^C NMR spectra (whole HSP sheet) and **c** ^1^H NMR spectra in D_2_O (Ir catalyst).
